# Face-body integration of intense emotional expressions of victory and defeat

**DOI:** 10.1371/journal.pone.0171656

**Published:** 2017-02-28

**Authors:** Lili Wang, Lisheng Xia, Dandan Zhang

**Affiliations:** 1 School of Educational Science, Huaiyin Normal University, Huaian, China; 2 Institute of Affective and Social Neuroscience, Shenzhen University, Shenzhen, China; 3 State Key Laboratory of Cognitive Neuroscience and Learning, Beijing Normal University, Beijing, China; Universita degli Studi di Udine, ITALY

## Abstract

Human facial expressions can be recognized rapidly and effortlessly. However, for intense emotions from real life, positive and negative facial expressions are difficult to discriminate and the judgment of facial expressions is biased towards simultaneously perceived body expressions. This study employed event-related potentials (ERPs) to investigate the neural dynamics involved in the integration of emotional signals from facial and body expressions of victory and defeat. Emotional expressions of professional players were used to create pictures of face-body compounds, with either matched or mismatched emotional expressions in faces and bodies. Behavioral results showed that congruent emotional information of face and body facilitated the recognition of facial expressions. ERP data revealed larger P1 amplitudes for incongruent compared to congruent stimuli. Also, a main effect of body valence on the P1 was observed, with enhanced amplitudes for the stimuli with losing compared to winning bodies. The main effect of body expression was also observed in N170 and N2, with winning bodies producing larger N170/N2 amplitudes. In the later stage, a significant interaction of congruence by body valence was found on the P3 component. Winning bodies elicited lager P3 amplitudes than losing bodies did when face and body conveyed congruent emotional signals. Beyond the knowledge based on prototypical facial and body expressions, the results of this study facilitate us to understand the complexity of emotion evaluation and categorization out of laboratory.

## Introduction

For humans, both face and body play important roles in conveying emotional information. Neuroscience studies consistently reveal that the brain detects facial and body expressions very rapidly [1]. In particular, it is found that the amygdala begins to respond to fearful faces as early as 40 ms after stimulus onset [2, 3]; and the event-related potential (ERP) components of lateral occipital P1 peaks at approximately 100–120 ms after emotional faces [4–8] and bodies [9] being displayed. In addition, studies have demonstrated that the early P1 also reflects the effect of emotion, usually with enhanced amplitudes in response to fearful compared to happy, sad, and neutral faces [7, 10] and bodies [9].

In everyday social interaction, face and body work as an integrated whole to make sure the emotional information being delivered efficiently and reliably. Figuring out how facial and body expressions are integrated and how face-body compound influences emotion perception and recognition constitute one of the most important issues in affective neuroscience. Previous studies usually employed prototypical facial and body expressions (e.g., NimStim Set of Facial Expressions [11], Bochum Emotional Stimulus Set [12], Body Expressive Action Stimulus Test [13, 14], and Chinese Affective Face Picture System [15]) and revealed that the judgment for facial expressions is largely influenced by body expressions. In particular, when the emotion category of body postures is mismatched with that of faces, the category accuracy for facial emotion is significantly reduced and subjects' judgment of facial expression is biased toward the emotion category expressed by the body [13, 16–20]. Importantly, while these studies (using posed prototypical face-body pictures) revealed a < 10% accuracy enhancement of facial expressions when body information was added, Aviezer et al. [21, 22] suggested that emotional body context is indispensable for recognition of intense real-life facial expressions. In Aviezer's study [22], participants were required to evaluate the valence of the pictures of professional tennis players, who were photographed when winning or losing a point in international tournaments. Results showed that when ratings were based on face-body compounds, or bodies alone, the pictures of winners and losers were accurately differentiated; however, when ratings were based on faces alone, the faces of winners and losers were totally undistinguishable.

Intense or extreme emotions often appear when something unusual happens, e.g., in the face of a car accident, winning a lottery with a huge prize, or losing a point on the last stage of a match at the Olympic Games. In general, emotions with relatively high intensity are believed to be better discriminated than those with low intensity because the formers activate more diagnostic action units in the face [23] and they are located on more extreme positions on the pleasure-displeasure axis [24]. However, perception of extremely intense emotions from facial expressions may be difficult in real-life situations, where facial behaviors are more complex and may involve a variety of non-typical expressions. For example, the defeated players in sport competition often exhibit a complex expression of sadness, regret, contempt, disgust, and even fear, which signals a distinct but undifferentiated negative emotional state [25, 26]. So far as we know, studies focused on the integration of intense facial and body emotions are largely insufficient.

The ERP technique is an ideal method to examine the time course of the integration of emotional signals from faces and bodies. To date, there are two ERP studies focusing on this topic [16, 19]. It was found that the occipital P1 was the earliest component related to the integration process, with a larger P1 for incongruent relative to congruent face-body compound stimuli [16]. Also, enhanced P1 amplitudes were observed when participants viewed face-body images with fearful bodies compared to happy bodies [19]. In the two studies, participants were required to focus on faces and judge facial emotions. The results suggested that body expressions can be processed rapidly, even when they are unattended in the task. The second informative component is the N170, which is measured over the occipito-temporal regions and is thought to reflect the structural encoding stage of faces [4, 6, 7, 27]. Similar to the face-evoked N170, bodies also elicit a N190 component, which peaks later than N170 [28]. The face-inversion effect on the N170 has also been found for bodies [29, 30], with larger N190 amplitudes/latencies for inverted than upright bodies. Moreover, the N190 could be modulated by emotional information as well as implicit motions conveyed by bodies [31, 32]. Furthermore, Gu et al. [19] found that the N2 component, as an index of conflict detection, was enhanced for incongruent than congruent face-body compounds. Meanwhile, researchers found a larger P3 for images with congruent, compared to incongruent, emotional signals [19], suggesting that the brain allocates more sustained attention to elaborately process the emotional information contained in congruent (rather than incongruent) face-body compound stimuli.

The aim of the present study was to investigate the time course of the integration of emotional signals from intense real-life face and body pairs. Emotional expressions of professional players (tennis, table tennis and badminton) reacting to winning or losing a point in tournaments were employed in the study. Compared to the prototypical expressions used in most of the previous studies, the pictures of winners and losers were not posed and they reflected natural responses to extreme situations. We investigated different stages of integrated processing of faces and bodies, as indexed by four ERP components (P1, N170, N2, and P3). According to previous studies [16, 19, 33, 34], the P1 is thought to reflect attention allocation in the early stage of visual processing; the N170 is related to the structural encoding of the compound face-body stimulus; the N2 is sensitive to conflict detection, and the P3 reflects sustained selective attention to motivationally relevant stimuli. Given that the human brain rapidly detects emotional signals, we predict that the integration of the two emotional signals starts early and the congruence effect may occur within the time window of the P1 component. Especially, body expression is important for the valence judgment of facial expressions, and it is possible that the main effect of body expression would be observed in multiple stages of integration.

## Method

### Participants

Twenty-one healthy undergraduates (13 females; age range = 19–23 years) participated in the ERP study. A different sample of fifteen undergraduates (7 females; age range = 21–25 years) participated in the behavioral rating of valence and intensity of pictures. Another sample of nineteen undergraduates (10 females; age range = 18–21 years) participated in the behavioral identification of the type of facial emotions. All subjects were right-handed and had normal or corrected-to-normal vision. Written informed consent was obtained prior to the experiment. The experimental protocol was approved by the local ethics committee (Shenzhen University) and this study was performed strictly in accordance with the approved guidelines.

### Stimuli

**Fig 1:**
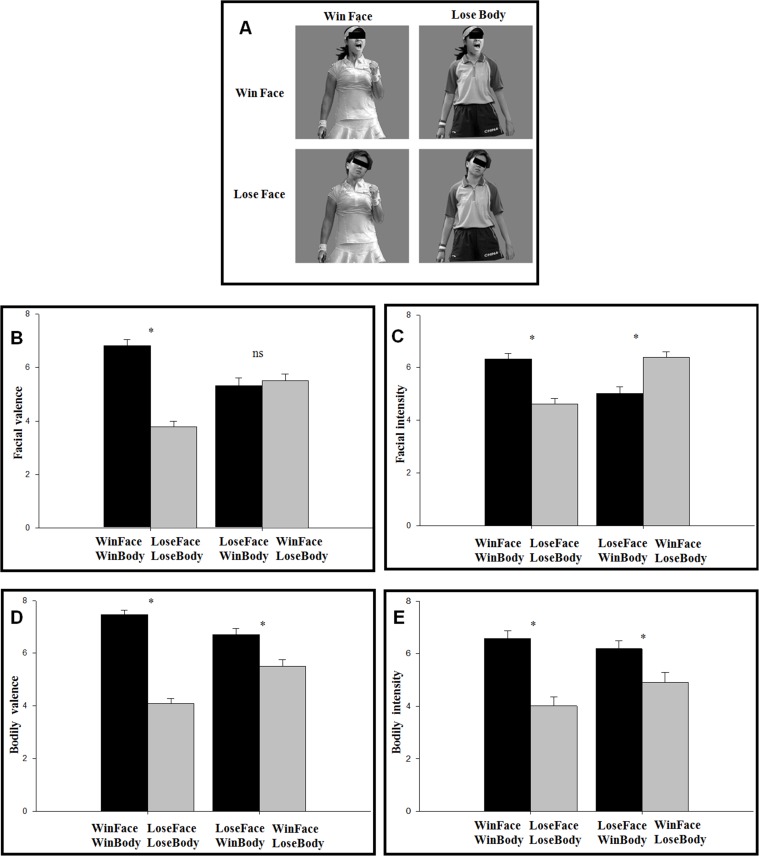
(**A**) Examples of experimental stimuli. (**B**) The valence of facial expressions (9-point scale, 1-the most negative, 9-the most positive). (**C**) The intensity of facial expressions (9-point scale, 1-the least intense, 9-the most intense). (D) The valence of body expressions. (E) The intensity of body expressions. Asterisk indicates significant difference (*p* < 0.05) while ns indicates no significance. Error bars show one standard error (S.E).

According to Aviezer et al.[22], 60 images (30 winners and 30 losers) were obtained through Google and Baidu image search, using the search keyword “reacting to winning a point” or “reacting to losing a point”, intersected with “tennis” or “table tennis” or “badminton”. Gender distribution was equal. Among the 60 original images, 54 ones had a front view while the other 6 images had a side view (but not exceeding 90°). Face–body compound stimuli were created using Photoshop software. Besides the 60 original images (congruent condition), another 60 “identities” were created by cropping and randomly cross planting the faces to bodies (incongruent condition). When producing new images, we ensured that the newly created face-body compounds look in a realistic manner [22]. Thus four image categories were made, i.e., 30 "congruent" winner's pictures, 30 "congruent" loser's pictures, 30 "incongruent" pictures with winner's face and loser's body, and 30 "incongruent" pictures with winner's body an loser's face. All stimuli were gray-scale photographs on the gray background with the same contrast and brightness (5.7°× 5.7° visual angle). The face–body ratio was fixed into approximately 1:3 for all pictures. Examples of the stimuli are displayed in Fig 1A.

### Procedure

**Fig 2:**
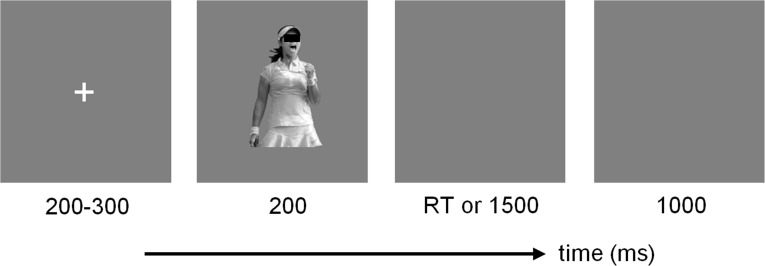
Schematic diagram of one experimental trial.

Stimuli were presented on a LCD monitor (refresh rate = 60 Hz). Each face-body compound stimulus was presented twice. The ERP experiment consisted of three blocks, each containing 80 trials. The order of the three blocks was pseudo-randomized across subjects. Blocks were separated by self-terminated breaks. As shown in Fig 2, each trial started with a 200-to-300-ms fixation, followed by a 200-ms face-body image. Participants were instructed to keep their eyes fixed on the fixation and indicate the valence (negative or positive) of the facial expression as quickly and accurately as possible, by pressing the “F” or “J” key with their left or right index finger. Key-response mappings were counterbalanced across participants. The response screen would not disappear until a button press or until 1500 ms elapsed.

In the picture rating task, subjects rated the valence and intensity of facial and body expressions on the 9 point scale [22] (valence: 1-the most negative and 9-the most positive; intensity: 1-the least intense and 9-the most intense). The facial and body expressions were rated in separate blocks. When rating the intensity, subjects were required to evaluate the muscular activity in faces or bodies, irrespective of the valence. All the 120 face-body pictures in the ERP experiment were presented in a random order. Pictures displayed on the screen until a response was made.

Additionally, a behavioral task was conducted to determine the type of emotion (neutral, happiness, sadness, anger, fear, and disgust) expressed by faces. All the 120 face-body pictures were presented in a random order. Subjects were asked to identify the facial expression by pressing 1 to 6 on the keyboard. Pictures displayed on the screen until a response was made.

### Electrophysiological recording and analysis

Brain electrical activity was recorded referentially against left mastoid and offline re-referenced to the global average reference, by a 64-channel amplifier with a sampling frequency of 250 Hz (Brain Products, Gilching, Germany). The electrode impedance was maintained below 5 kΩ. Horizontal electrooculographies (EOGs) were recorded from two electrodes at the outer chathi of each eye. Vertical EOGs were recorded from electrodes situated on infraorbital and supra-orbital regions of the right eye. The experimenter monitored the EOG data online to ensure that participants did not move their eyes between the fixation and the face.

Ocular artifacts were removed from EEGs by using the method proposed by Gratton and Coles [35], as implemented in the Brain Vision Analysis software (Version 2.0). The EEG data were filtered within 0.05–30 Hz. Trials contaminated with larger artifacts (exceeding ± 50 μV) were excluded from the averaging (approximately 10 trials per individual were rejected). The mean number of trials included in the ERP averaging was 49, 49, 50, and 50, for the four conditions (Win Face-Win Body, Lose Face-Lose Body, Win Face-Lose Body, and Lose Face-Win Body). The EEG data were segmented beginning 200 ms prior to the onset of targets and lasting for 1000 ms. All epochs were baseline-corrected with respect to the mean voltage over the 200 ms preceding the onset of targets, followed by averaging in association with experimental conditions irrespective of response [16, 19].

Four ERP components (P1, N170, N2 and P3) were analyzed in this study. The P1, N170 and N2 were measured by peak amplitude and peak latency. The time windows for the P1, N170 and N2 were 70–130 ms, 100–200 and 150–350 ms respectively. The P1 was analyzed at the electrode sites of Oz, O1, O2, PO7 and PO8. The N170 was analyzed at P7 and P8. The N2 was analyzed at FCz, FC1, FC2, Fz, F1, and F2. The mean amplitude of P3 was calculated at the electrode sites of P1, Pz, and P2 (time window = 350–550 ms, mean peak latency = 450 ms). Three-way repeated measures analyses of variance (ANOVA) were conducted with body expression (positive and negative), congruence (congruent and incongruent) and electrode (five levels for P1: Oz, O1, O2, PO7 and PO8; two levels for N170: P7 and P8; six levels for N2: FCz, FC1, FC2, Fz, F1, and F2; and three levels for P3: P1, Pz, and P2) as the three within-subjects factors. Greenhouse-Geisser correction for ANOVA tests was used whenever appropriate [36]. Partial eta-squared (η_p_^2^) was reported to demonstrate the effect size in the ANOVA tests. Bonferroni correction was used for multiple comparisons in the statistical analysis of ERP data.

## Results

### Picture rating

Two-way repeated measures ANOVA was performed, with body or face expression (positive and negative) and congruence (congruent and incongruent) as two within-subject factors.

For the valence rating of faces (Fig 1B), there was a significant main effect of body expression [*F*(1,14) = 45.8; *p* < 0.001; *η*_*p*_^*2*^ = 0.766]. The facial valence was higher for the pictures with winning (M ± SE, 6.07 ± 0.27) compared to losing bodies (4.65 ± 0.17). The body expression × congruence interaction was significant [*F*(1,14) = 118; *p* < 0.001; *η*_*p*_^*2*^ = 0.894]. The valence was higher for winning (6.82 ± 0.23) than for losing bodies (3.78 ± 0.21, *p* < 0.001) in the congruent condition, but there was no difference between winning (5.32 ± 0.31) and losing bodies (5.51 ± 0.25, *p* = 0.160) in the incongruent condition.

For the intensity rating of faces (Fig 1C), the body expression × congruence interaction was significant [*F*(1,14) = 51.9; *p* < 0.001; *η*_*p*_^*2*^ = 0.787]. The facial intensity was higher for winning (6.32 ± 0.23) than for losing bodies (4.62 ± 0.20, *p* < 0.001) in the congruent condition, but the intensity was higher for losing (6.39 ± 0.21) than for winning bodies (5.03 ± 0.26, *p* < 0.001) in the incongruent condition. In other words, the intensity was higher for the compounds with winning (6.35 ± 0.21) compared to losing faces (4.82 ± 0.22).

For the valence rating of bodies (Fig 1D), there was a significant main effect of body expression [*F*(1,14) = 100; *p* < 0.001; *η*_*p*_^*2*^ = 0.878]. The valence of the body was higher for the pictures with winning (7.08 ± 0.20) compared to losing bodies (4.80 ± 0.19). The body expression × congruence interaction was significant [*F*(1,14) = 35.4; *p* < 0.001; *η*_*p*_^*2*^ = 0.716]. The difference between winning (7.46 ± 0.18) and losing bodies (4.10 ± 0.18, *p* < 0.001) in the congruent condition was larger than the difference between winning (6.70 ± 0.24) and losing bodies (5.51 ± 0.26, *p* = 0.004) in the incongruent condition.

For the intensity rating of bodies (Fig 1E), there was a significant main effect of body expression [*F*(1,14) = 54.6; *p* < 0.001; *η*_*p*_^*2*^ = 0.796]. The intensity of the body was higher for the pictures with winning (6.38 ± 0.29) compared to losing bodies (4.46 ± 0.34). The body expression × congruence interaction was significant [*F*(1,14) = 15.3; *p* = 0.002; *η*_*p*_^*2*^ = 0.522]. The difference between winning (6.58 ± 0.28) and losing bodies (4.01 ± 0.34, *p* < 0.001) in the congruent condition was larger than the difference between winning (6.19 ± 0.31) and losing bodies (4.91 ± 0.37, *p* = 0.003) in the incongruent condition.

**Fig 3:**
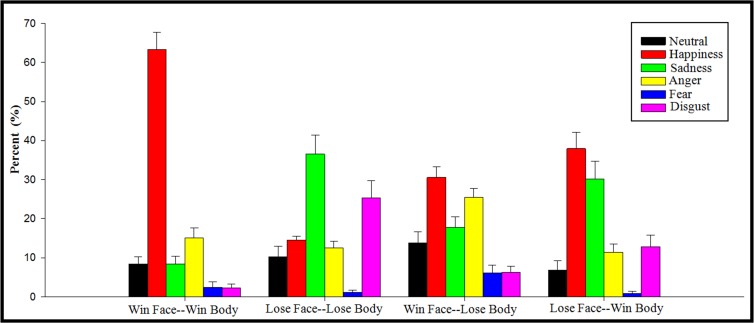
Rating for the type of emotion expressed by faces in different face-body conditions.

For the behavioral identification of facial expressions, we first computed chi-square values of the four categories of pictures, using the type of expression (neutral, happiness, sadness, anger, fear, and disgust) as the independent variable and the identification percentage of each emotion as the dependent variable (Table 1). All the four Chi-squares were significant (*p* < 0.001; Fig 3). Then the recognition rate of each expression type was compared with the chance level (1/6). The result of one-sample t-test showed that 1) for the condition of Win Face-Win Body, happiness (63%; *t*(18) = 10.6, *p* < 0.001) was identified most frequently; 2) for the condition of Lose Face-Lose Body, sadness (36%; *t*(18) = 4.07, *p* = 0.001) was identified most frequently; 3) for the condition of Win Face-Lose Body, happiness (30%; *t*(18) = 5.13, *p* < 0.001) and anger (25%; *t*(18) = 3.73, *p* = 0.002) were identified most frequently; and 4) for the condition of Lose Face-Win Body, happiness (38%; *t*(18) = 5.07, *p* < 0.001) and sadness (30%; *t*(18) = 2.98, *p* = 0.008) were identified most frequently.

**Table 1:** Rating for the type of emotion expressed by faces (data are reported using percent (%) and presented as mean ± SD).

Picture Condition	Neutral	Happiness	Sadness	Anger	Fear	Disgust	χ^2^ *df =* 5	*P*
Win Face Win Body	8.4 ± 7.6	63.3 ± 19.2	8.4 ± 8.6	15.1 ± 11.1	2.5 ± 5.8	2.3 ± 4.4	167.1	< 0.001
Lose Face Lose Body	10.2 ± 12.2	14.6 ± 4.2	36.5 ± 21.2	12.5 ± 7.8	1.0 ± 2.7	25.3 ± 19.2	45.9	< 0.001
Win Face Lose Body	13.9 ± 2.3	30.5 ± 1.8	17.7 ± 2.0	25.4 ± 0.3	6.1 ± 8.8	6.3 ± 6.3	61.0	< 0.001
Lose Face Win Body	6.8 ± 10.3	37.9 ± 18.3	30.2 ± 19.8	11.4 ± 8.9	0.9 ± 2.4	12.8 ± 13.0	30.7	< 0.001

### Accuracy rate (ACC)

For the ERP experiment, the main effect of congruence was significant [*F*(1,20) = 43.8; *p* < 0.001; *η*_*p*_^*2*^ = 0.687]. The ACC was higher for congruent (75.7 ± 2.3%) than for incongruent images (52.5 ± 2.2%). Moreover, the discrimination performance of incongruent trials was at chance level (one-sample t-test, compared with 50%, *p* > 0.05).

### Reaction time (RT)

There was a significant main effect of congruence [*F*(1,20) = 9.00; *p* = 0.007; *η*_*p*_^*2*^ = 0.309]. Subjects responded faster in the congruent condition (479.4 ± 31.8 ms) than in the incongruent condition (496.0 ± 33.7 ms).

The ACC and RT results are shown in Table 2.

**Table 2:** Behavioral results, face-body compound stimuli (data are presented as mean ± SD).

	Congruent		Incongruent	
Emotion condition	Win Face Win Body	Lose Face Lose Body	Win Face Lose Body	Lose Face Win Body
Accuracy, %	81.6 ± 13.5	69.8 ± 18.2	55.9 ± 19.9	49.2 ± 23.4
Reaction time, ms	461.5 ± 148.9	497.3 ± 154.0	504.2 ± 168.6	487.62 ± 147.2

### P1

#### Peak amplitude

**Fig 4:**
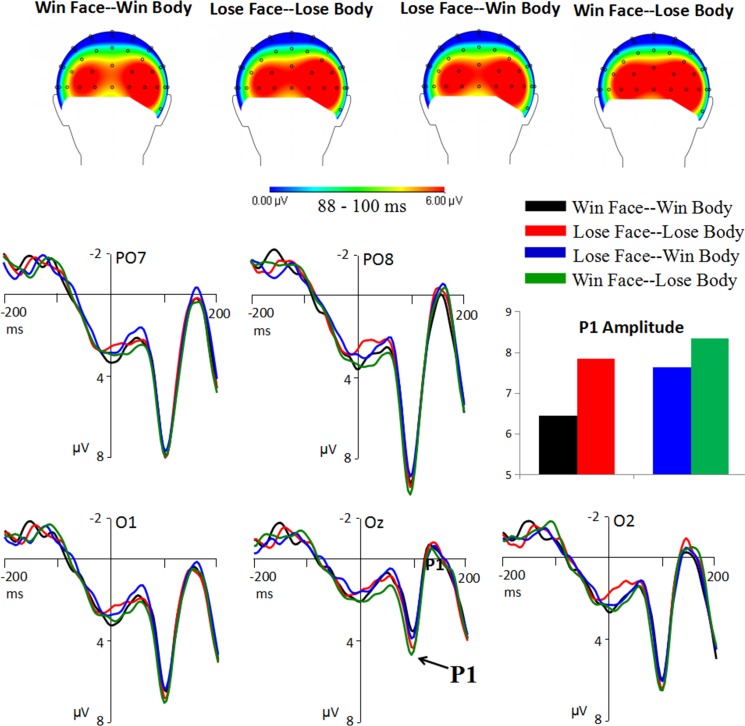
Grand average of the P1 component. Please note that the topographic maps did not show the distribution of voltage over the left mastoid (i.e. the reference electrode).

There was a significant main effect of electrode [*F*(4,80) = 23.2; *p* < 0.001; *η*_*p*_^*2*^ = 0.537], with larger amplitudes at the occipito-parietal sites (PO7 = 8.67 ± 0.94 μV, PO8 = 9.88 ± 0.93 μV) than at the occipital sites (Oz = 5.05 ± 0.87 μV, O1 = 7.20 ± 0.91 μV, O2 = 7.05 ± 0.85 μV; *ps* < 0.05). There was also a main effect of body expression [*F*(1,20) = 16.8; *p* = 0.001; *η*_*p*_^*2*^ = 0.457]. As shown in Fig 4, the P1 was larger following losing (8.10 ± 0.88 μV) compared to winning bodies (7.05 ± 0.81 μV). A main effect of congruence was also found [*F*(1,20) = 25.17; *p* < 0.001; *η*_*p*_^*2*^ = 0.557], with enhanced P1 amplitudes in response to incongruent (8.00. ± 0.86 μV) compared to congruent (7.16 ± 0.82 μV) face-body compounds.

#### Peak latency

No main effect or interaction was significant. The latency for the four conditions (Win Face-Win Body, Lose Face-Lose Body, Win Face-Lose Body, and Lose Face-Win Body) was 96.9 ± 1.65 ms, 97.3 ± 1.65 ms, 96.0 ± 1.52 ms and 98.3 ± 2.06 ms, respectively.

### N170

#### Peak amplitude

**Fig 5:**
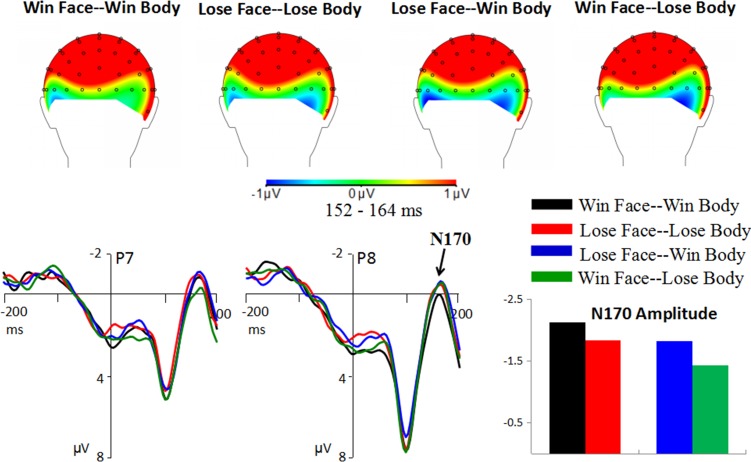
Grand average of the N170.

There was a significant main effect of body expression [*F*(1,20) = 6.77; *p* = 0.017; *η*_*p*_^*2*^ = 0.253]. As shown in Fig 5, enhanced N170 amplitudes were observed in response to the stimuli with winning (-1.97 ± 0.50 μV) compared to losing bodies (-1.64 ± 0.47 μV).

#### Peak latency

There was a significant main effect of congruence [*F*(1,20) = 6.28; *p* = 0.021; *η*_*p*_^*2*^ = 0.239]. Congruent face-body compounds (155.33 ± 2.20 ms) elicited faster responses than incongruent compounds did (160.29 ± 2.263 ms).

### N200

#### Peak amplitude

**Fig 6:**
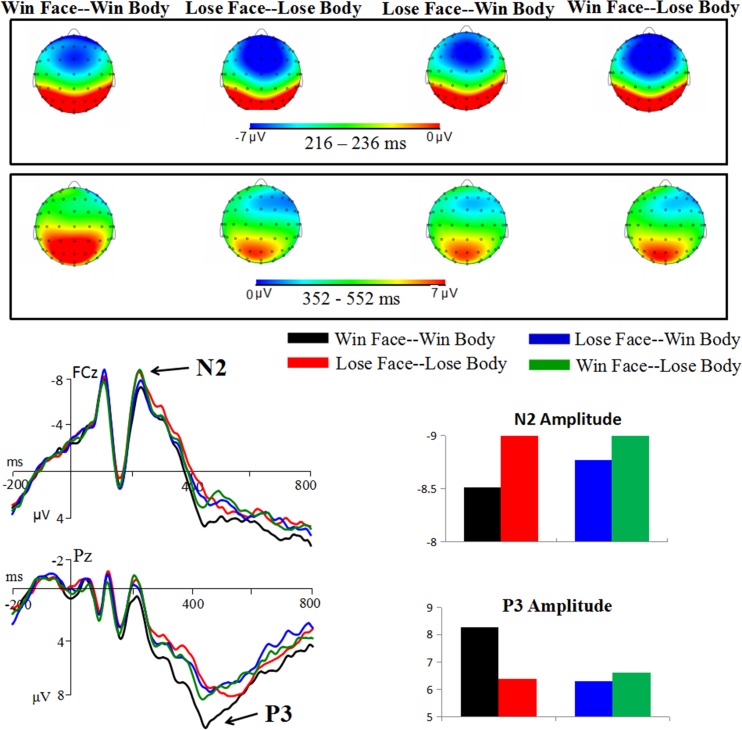
Grand average of the N2 and P3.

There was a significant main effect of body expression [*F*(1,20) = 7.34; *p* = 0.013; *η*_*p*_^*2*^ = 0.269]. As shown in Fig 6, enhanced N2 amplitudes were observed in response to the stimuli with losing (-9.35 ± 1.27 μV) compared to winning bodies (-8.64 ± 1.24 μV).

#### Peak latency

No main effects or interactions were significant. The latency for the four conditions (Win Face-Win Body, Lose Face-Lose Body, Win Face-Lose Body, and Lose Face-Win Body) was 253 ± 9.34 ms, 248 ± 8.67 ms, 244 ± 9.54 ms and 246 ± 8.27 ms, respectively.

### P3

ANOVAs for the P3 amplitudes showed a significant main effect of congruence [*F*(1,20) = 11.2; *p* = 0.003; *η*_*p*_^*2*^ = 0.360], with enhanced P3 amplitudes in response to congruent (7.33 ± 0.64 μV) compared to incongruent (6.46 ± 0.66 μV) face-body compounds.

More importantly, the interaction effect of congruence by body expression was significant [*F*(1,20) = 7.02; *p* = 0.015; *η*_*p*_^*2*^ = 0.260]. The stimuli with winning bodies (8.27 ± 0.75 μV) elicited larger P3 amplitudes than the stimuli with losing bodies (6.39 ± 0.76 μV) in the congruent condition (*p* = 0.021). However, there was no difference between winning (6.30 ± 0.62 μV) and losing (6.63 ± 0.77 μV) bodies in the incongruent condition (*p* = 0.435). In addition, the scalp distribution of the P3 (Fig 6) was wider for the condition of Win Face-Win Body than the other three conditions.

## Discussion

In this study, we focused on the emotional processing of intense face-body expressions and investigated the influence of emotional body language on the processing of emotional faces. The behavioral results showed that the judgment was faster and more accurate for congruent than for incongruent face-body compounds. Also, the facial valence was rated as more positive when faces were accompanied by congruent winning bodies. Our result is in agreement with the idea that body context influences the recognition of facial expressions, i.e., congruent emotional body language facilitates the recognition of facial expressions while conflicting body expression hampers the latter [16]. At the same time, our ERP data showed that the body expression influenced different temporal stages of facial expression processing, as evidenced by the P1, N170, N2 and P3 components.

### The P1 reflects a negative bias towards losing bodies

At the early processing stage of primary integration of emotional signals from faces and bodies, the face-body compounds with losing, compared to wining, bodies elicited larger P1 amplitudes over the parieto-occipital areas. In our opinion, the P1 result might reflect a negativity bias, which is defined as an attention bias towards negative stimuli [37, 38]. The P1 is considered to reflect early attention allocation in the extrastriate cortex [16], and enhanced P1 amplitudes have been reported in response to emotionally negative stimuli (e.g. fearful faces and bodies, and negative words) when compared with neutral or positive stimuli [5–7, 9, 10]. Additionally, the P1 in this study reached maximum at the parieto-occipital sites (i.e. PO7 and PO8), which is different from its typical occipital topography [16, 19]. This result may be because the stimuli of this study was different from the prototypical expressions employed in previous EEG studies [16, 19], which were highly recognizable [21] and signaled emotions clearly. The parieto-occipital topography of P1 implies that the processing of emotions with extremely high intensity might involve more brain regions besides the primary visual cortex.

The finding of P1 provided evidence for an early negative bias towards negative body expressions. In line with this finding, two previous ERP studies found larger P1 amplitudes for fearful relative to neutral [9] and happy bodies [19]. Recently, Borgomaneri et al. found the influence of such negative bias on the motor system [39, 40]. Moreover, a recent study by Meeren et al. [41] showed rapid parietal responses to fearful bodies as early as 80 ms after stimulus onset, suggesting a fast emotion-attention-action link in the dorsal visual stream.

An alternative explanation is that bodies may be easier to get attention than faces. In general, body expressions allow humans to signal emotions to others over a large distance because the emotional information is mainly contained in the outlines of bodies [42]. On the contrary, the emotion conveyed by faces is largely contained in facial details (e.g., mouth, eyebrow and eye). In the current study, the face-body compound was presented briefly (200 ms), requiring participants to make a decision on the basis of their “first impression”, so global rather than fine-grained analysis was more crucial for the task [16]. Moreover, the fixation point was placed at the bottom of breast, thus it may be easier for participants to automatically perceive the emotional information of bodies. More importantly, the current study used face-body pictures with extremely intense emotions, thus the face was likely to provide highly ambiguous information on valence[22]. Therefore, participants might rely more on bodies than faces to discriminate valence at the early processing stage, even in the case where the body information was task-irrelevant.

### The emotional congruence effect at different stages of face-body integration

At the early stage of integration, enhanced P1 was observed for incongruent than congruent face-body compounds, suggesting that the evaluation of the relation between faces and bodies took place very early, and that incongruent face-body compounds may attract more attention than the congruent compounds at this processing stage. When using the prototypical expressions, Meeren, et al [16] also found the congruence effect on the P1.

The discrimination performance for the incongruent trials was at chance level (52.5%), indicating that the conflicting body expressions hampered the recognition of facial expressions. This idea is also consistent with the rating result, i.e., no difference was found between winning and losing faces in the incongruent condition. Besides the influence of incongruent body expression, the low accuracy for the incongruent trials might be partly due to the location of the fixation point, approximately placing at the bottom of breast and above belly. It is possible that the fixation point on the body made it difficult to recognize the facial expression in the rapid presentation (200 ms) [19]. For example, when the fixation was on the face, the recognition accuracy in incongruent trials was 85% [19]; however, when the fixation was on the body, the accuracy decreased to 67% [16] (Note that both the two studies used postured emotional pictures so their results are comparable).

In addition, the muscular activity of faces was also a discriminative measure for the reorganization of face-body compounds. We found higher muscular intensities in winning compared to losing faces (Fig 1C), which was in line with Aviezer et al [22]. According to the analysis of facial movements [21], winning faces often displays more activity (e.g. mouth opening, smiling) than losing faces. Therefore, both faces and bodies provide irreplaceable emotional information, i.e., faces provide intensity information and bodies provide valence information.

We also found the congruence effect on N170 latency, with a delayed N170 for the incongruent face-body compounds. The N170 is thought to reflect the structural encoding of emotional faces/bodies, and a delayed N170 was often found for inverted, compared to upright, faces and bodies [29, 30]. The explanation of the inversion effect is that inverted stimuli alter the prototypical spatial relations between face/body parts [43] and disrupt the processing of configurational information [30, 44]. In the current study, the latency difference between congruent and incongruent compounds indicates that the incongruent emotion between the face and the body destroys the normal configuration of face-body compounds.

In the later stage of face-body integration, the present study found a main effect of congruence on the P3 (congruence > incongruence). The P3 component is thought to represent stimulus evaluation and categorization [45, 46], and it increases with the amount of effort devoted to a task [47]. The P3 was larger in the congruent relative to the incongruent condition, suggesting that participants devoted greater effort to evaluate the congruent face-body compound. In the study of Gu et al. [19], a larger P3 was also found for images with congruent than incongruent emotional signals. In another cross-modal study, more neural activity in the amygdala was observed when fearful faces were paired with congruent emotional voices [48].

Moreover, a significant interaction of congruence by body valence on the P3 component was observed. Winning bodies elicited lager P3 amplitudes than losing bodies only for congruent images. In line with our finding, Olofsson et al. [49] reviewed a series of ERP studies involving the processing of emotional pictures, and concluded that pleasant stimuli usually elicit a larger P3 than unpleasant stimuli [50, 51]. Besides the valence effect of P3, an alternative explanation was the uncertainty effect, which means that the P3 amplitude would decrease when the subject is uncertain of the category of stimuli [52]. Compared with the thrill of victors, the defeated players often exhibit a complex expression of sadness, regret, contempt, disgust, and even fear [25]. In line with this proposal, the subjective identification of facial emotions showed that the winning pictures were associated with a higher certainty (i.e., 63% pictures were identified as happiness) for categorical decision-making [53, 54], as compared to losing pictures [25]. Also, the behavioral result showed that winning pictures were associated with higher accuracy (81.6%) than losing pictures (69.8%).

### The effect of body expression on N170 and N2

In the current study, we observed the main effect of body expression on the amplitudes of N170 and N2 components. Interestingly, our finding that larger N170 amplitudes for winning compared to losing bodies is different from previous studies, which demonstrated that the N170 (or body-related N190) was larger for fearful compared to happy bodies [31, 32]. It was found that both motion and emotion could modulate the N170/N190. The study by Borhani et al. [31, 32] suggested that when fearful and happy bodies both convey a large amount of motion information, the emotion factor would affect the N170/190, thus eliciting larger amplitudes for fearful compared to happy postures. However, the pictures used in this study did not have comparable motion information between winning and losing bodies. That is, winners often display an expanded body posture (e.g., with arms raised above the shoulder, or a clenched fist), expressing an emotion of triumph [55]. In comparison, losers usually display diminutive body postures (e.g., drooping arms). Therefore, it is possible that the N170 finding of this study is caused by the motion of body postures. Further studies are needed to analyze the muscle activations of winners and losers according to the Body Action Coding System [56]. The N170 finding implies that the structural encoding involves not only a perceptual representation of the body, but also a more detailed analysis of emotion and action conveyed by body postures [32].

In the study using the prototypical expressions, Gu et al. [19] found larger N2 amplitudes in the incongruent condition, which was the earliest congruence effect in that study. However, this study as well as the study by Meeren et al.[16] found the earliest congruence effect on the P1 component. The inconsistency between studies may be due to the differences in the location of fixation point, which was on face in Gu et al [19] and on the chest in this study and Meeren et al. [16]. The fixation on the chest may result in faster detection of the conflict between facial and body emotions.

Finally, two limitations should be pointed out for an appropriate interpretation of the current result. First, participants were asked to judge the facial expressions, irrespective of the information transferred by the bodies. Although the body expression was task-irrelevant, we observed the influence of emotional body at different stages of integration. As suggested by Gu et al [19], the focus of attention (face or body) modulates differently the early and late stages of face-body integration. For example, they found a larger P3 for fearful compared to happy bodies only when attention was focused on the body. Therefore, future studies should include another experiment block, asking participants to judge the body expression, irrespective of the information transferred by the faces. Second, this study recruited different subjects to participate the behaviorally picture rating task and the ERP task, which prevents us to make direct inference on the relation between ERP and subjective rating results.

In conclusion, the present study explored intense expressions of face-body pictures in real-life scenarios (i.e. with high ecological validity). We found that the recognition of facial expressions was highly dependent on the associated body emotions. The ERP findings suggest that the integration of emotional signals from faces and bodies takes place as early as 90–100 ms after the stimulus onset. We also observed the effect of body expression at different stages of integration, as index by P1, N170 and N2. And from 350–550 ms after stimulus onset, more sustained attention was allocated to the winning pictures. Beyond the knowledge based on prototypical facial and body expressions, the results of this study facilitate us to understand the complexity of emotion evaluation and categorization out of laboratory [25].
